# Sexual Identity and Self-Rated Health in Midlife: Evidence from the Health and Retirement Study

**DOI:** 10.1089/heq.2021.0034

**Published:** 2021-09-14

**Authors:** Hui Liu, Ning Hsieh, Wen-hua Lai

**Affiliations:** Department of Sociology, Michigan State University, East Lansing, Michigan, USA.

**Keywords:** midlife, bisexual, gay, lesbian, self-rated health

## Abstract

**Purpose:** This study examined health disparities among U.S. sexual minority people in midlife—a critical life course stage that is largely overlooked in the sexual minority health literature.

**Methods:** Data were drawn from the 2016 Health and Retirement Study. We restricted the analysis to respondents aged 50–65. The final sample consisted of 3623 respondents, including 3418 self-identified heterosexual individuals, 99 self-identified gay/lesbian individuals, 38 self-identified bisexual individuals, and 68 respondents who identified as “something else.” Ordinal logistic regression models were estimated to predict the odds of reporting better health.

**Results:** Bisexual midlifers reported significantly worse health than their heterosexual counterparts after age, gender, and race-ethnicity are controlled for (OR=0.43, 95% CI=0.25–0.76); this health disparity is mostly explained by marital status, socioeconomic status, and health behaviors (in particular smoking and exercising). We did not find evidence of a self-rated health disadvantage among gay and lesbian midlifers relative to their heterosexual counterparts.

**Conclusion:** These findings highlight the diversity of the sexual minority population in midlife. Public policies and programs should be designed and implemented at the interpersonal and institutional levels to eliminate health and other social disadvantages among sexual minority people, in particular bisexual people, in midlife.

## Introduction

The public perception of lesbian, gay, and bisexual (LGB) people focuses largely on the young community, while LGB people who are middle-aged and older represent an understudied disadvantaged group. In the United States, more than 2.7 million adults aged 50 or older identify as LGB and this number is projected to reach 5 million by 2030.^1^ Midlife (which refers to ages 50–65 in this study) represents a pivotal period in the life course at the crossroads of youth and old age. Yet, previous studies of LGB health have devoted less attention to midlife than either the earlier or later periods of life.^2,3^

Midlife is often perceived as a period marked by stress and crisis typically due to experiences such as multiple role demands, empty nest syndrome, and the menopausal transition^2,4^; and LGB adults in midlife not only confront the same challenges that the general population of midlifers experience, but also face unique barriers and challenges (e.g., historical restriction from legal marriage) in maintaining good health due to their sexual minority status. This study is one of the first population-based analyses of health disparities by sexual identity among midlife adults using nationally representative data from the 2016 Health and Retirement Study (HRS).

### Prior empirical evidence

Previous studies on LGB health disparities with a specific focus on midlife are rare. Most prior studies of such disparities clump different age groups together without specific attention to midlifers—using a sample of young and middle-aged adults (e.g., ages 18–64) or middle-aged and older adults (e.g., ages 50 and older).^5–7^ A small number of initial studies have suggested that LGB people in midlife may have health experiences that differ from those of both their heterosexual counterparts and their older LGB counterparts.

However, the evidence is mixed likely due to differences in health outcomes examined, age ranges of analytic samples, and study designs.^6,8^ For example, an analysis of data from the National Health and Nutrition Examination Survey revealed that middle-aged LGB people (age 40–59), particularly those who identified as bisexual, reported higher levels of mental distress than their heterosexual counterparts, in part, because they had less social support.^8^ In contrast, a recent study based on data from the 2016 HRS showed that LGB respondents aged 50 and older reported better self-rated health than their heterosexual counterparts.^6^ However, this study did not distinguish bisexual and gay/lesbian respondents whose health experiences may differ.

An emerging number of studies have noted that bisexual people face marginalization in both the heterosexual and gay/lesbian communities (discussed in more detail below). Moreover, using data from the Caring and Aging with Pride study, Fredriksen-Goldsen et al. compared the experiences of LGB adults in different stages of the life course; the results indicated that young-old LGB adults (50–64) reported experiencing more lifetime victimization and discrimination and had a higher likelihood of substance use than two older groups of LGB adults (65–79 and 80+).^1^ Given the mixed and limited evidence on LGB health in midlife, additional research is clearly warranted.

### Health risk factors for LGB adults in midlife: a minority stress perspective

A prominent theoretical explanation of LGB people's challenges is minority stress theory, which suggests that due to the stigma associated with their sexual identity, including homophobia and biphobia, LGB people experience worse health outcomes than their heterosexual counterparts.^9,10^

#### Marital status

One of the most frequently documented sexual minority stressors is the historical restriction of same-sex marriage. In the United States, same-sex marriage was not legalized at the federal level until 2015. Therefore, a large number of middle-aged LGB adults were not able to legally marry their same-sex partners. In addition, due to social, cultural, and interpersonal stigma, midlife LGB adults face more challenges in the marriage market (e.g., finding a partner for marriage) than their heterosexual counterparts, and when they are able to marry, they may be more likely to experience a dissolution of marriage.^11–14^

Many empirical studies have demonstrated that married people have better health, both mental and physical, than unmarried people including cohabiting, divorced, widowed, and never married people.^15^ Marriage is found to be related to unique economic, social, and psychological resources that cannot be obtained from other types of relationships (such as cohabitation); these resources in turn affect health and well-being.^15–17^ For example, marriage leads to an increase in economic resources through specialization, economies of scale, and the pooling of wealth.^18^ Marriage also reinforces social integration by extending involvement in social relationships and by increasing access to social support and perceived security—all factors that may promote the health of married people.^15,16,19^ Thus, the higher proportion of LGB midlifers who are unmarried (relative to their heterosexual counterparts) may contribute to their poorer health.^20^

#### Socioeconomic status

Socioeconomic status (SES) is a fundamental cause of health and well-being.^21,22^ Although gay and lesbian people tend to have higher levels of education and income than their heterosexual counterparts, recent research shows that other sexual minorities, especially bisexual people, are among the most socioeconomically disadvantaged groups in the United States, with lower rates of educational attainment, higher rates of poverty and housing insecurity, and lower rates of health coverage than their straight counterparts.^23^ Such SES inequalities may lead to health disparities by sexual identity.

#### Health behaviors

Higher levels of stress exposure among sexual minority individuals promote risky health behaviors, which may be another potential mechanism leading to poorer health in midlife. A number of studies have found that sexual minority individuals are more likely to smoke and drink than their heterosexual counterparts.^24–26^ Although gay men engage in physical activity more often than heterosexual men (due, in part, to dissatisfaction with body image), lesbian women may be less physically active than heterosexual women.^24,27,28^ Because smoking, excessive drinking, and a sedentary lifestyle are all strong risk factors for poor health, these health behaviors may explain the poorer health of sexual minority individuals in midlife.

#### Unique stressors among bisexual people

Minority stress may not affect all middle-aged LGB adults equally. Notably, bisexual people may suffer greater stress due to marginalization in both the heterosexual and gay/lesbian communities and thus may experience more negative health consequences than their gay and lesbian counterparts.^29–31^ Hsieh and Liu found that married bisexual respondents, especially those in different-gender unions, exhibited poorer health than unmarried bisexual respondents; they argued that because bisexual people face unique stressors (e.g., doubts about their loyalty and commitment to a monogamous relationship), being in an intimate relationship may not entail the same health benefits for this group as for heterosexual, gay, and lesbian people.^30^ Moreover, prior research has found that bisexual people experience higher rates of unemployment, homelessness, and poverty than both heterosexual and gay/lesbian people.^7,24,29^ Thus, it is imperative to analyze bisexual people separately in assessments of the risk factors associated with sexual minority health in midlife.^32^

### Research hypotheses

*Hypothesis 1:* LGB people in midlife report worse self-rated health than their heterosexual counterparts, and this health disadvantage is larger for bisexual respondents than for gay or lesbian respondents.

*Hypothesis 2:* The self-rated health disadvantage of LGB adults in midlife is partially explained by marital status, SES, and health behaviors.

## Methods

### Data and sample

We used data from the HRS (2016), which was conducted by the Institute for Social Research at the University of Michigan. The HRS surveys a nationally representative sample of noninstitutionalized adults older than 50 years of age and their spouses.^33^ The survey oversamples black and Hispanic people and collects detailed information on cognitive, physical, economic, work, and family conditions, as well as health behaviors, approximately every 2 years (by telephone or in person). In 2016, the HRS collected sexual identity information for the first time among newly added refreshment respondents. Our study was based on the 2016 HRS despite its longitudinal design.

**FIG. 1.**
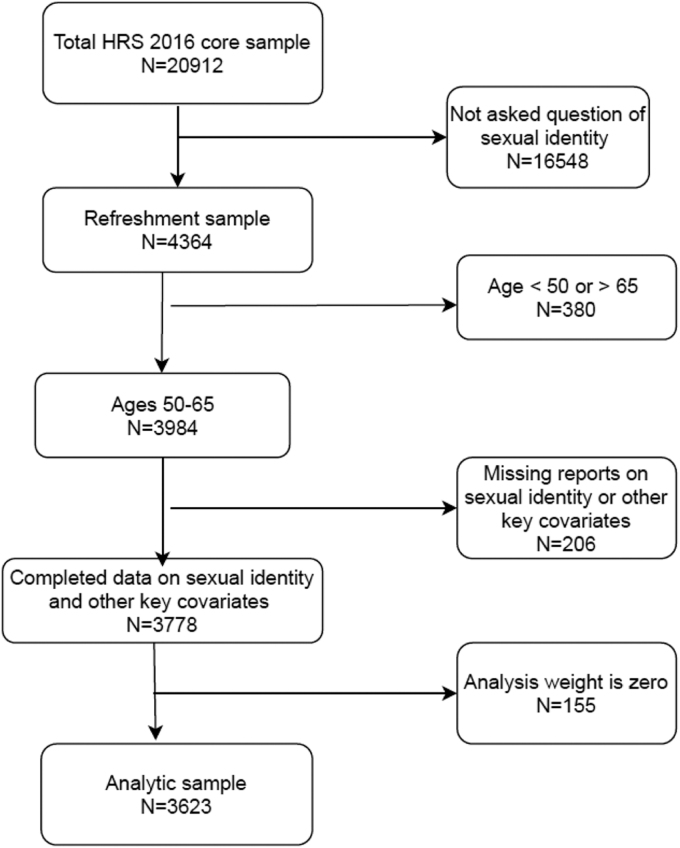
Flowchart of sample selection process.

We restricted the analysis to respondents aged 50–65 because no respondents older than 65 years identified as gay, lesbian, or bisexual. We excluded respondents with missing data on sexual identity or other key variables. The final sample consisted of 3623 respondents, including 3418 self-identified heterosexual individuals, 99 self-identified gay/lesbian individuals, 38 self-identified bisexual individuals, and 68 other nonheterosexual identified individuals. Figure 1 shows the flowchart of sample selection process.

### Measures

#### Dependent variable

*Self-rated health* was measured using the question: “Would you say your health is excellent, very good, good, fair, or poor?” Values were reverse coded so that higher values indicated better health. Self-rated health is a strong predictor for mortality, and the reliability and validity of the self-rated health measure is well-established.^34,35^

#### Independent variable

*Sexual identity* was measured based on the question: “Do you consider yourself to be lesbian/gay, straight, bisexual, or something else?” We coded four sexual identity categories: heterosexual (reference), lesbian/gay, bisexual, and other (i.e., “something else”).

#### Potential mediators

We considered marital status, SES, and health behaviors as potential explanatory variables driving differences in self-rated health across sexual identity groups in midlife. *Marital status* included four categories: married (reference), cohabiting, previously married (including divorced, separated, and widowed), and never married.

*SES* was indicated by education, household income, and household wealth. *Education* included four categories: less than high school (reference), high school graduate, some college, college graduate, and above. *Total household income* included the respondent's and the spouse's income from all sources such as earnings, pensions, and annuities, Supplemental Security Income and Social Security Disability, Social Security retirement, other government transfers, unemployment and workers' compensation, household capital income, and other income for the last calendar year before the survey. *Net household wealth* was measured as the total value of household assets minus household debts.

We used the RAND version of household income and wealth data, which includes consistently imputed missing values across waves.^36^ Because household income and wealth had zero or negative values, we added a constant of $1 for income and added a year-specific constant (depending on the minimum value of wealth in that specific year) for wealth for all respondents (so all wealth and income values were positive). We then divided the imputed income and wealth by the square root of household size to adjust for household size so that income of all households are on a comparable basis.^37^ We further took the natural logs of the values to adjust the skewed distribution of the variable.^37^

*Health behaviors* included smoking, drinking, and exercise. *Smoking* was measured by a dichotomous indicator: current nonsmoker (0) and current smoker (1). *Drinking* included three categories: current nondrinker (reference), current light drinker (fewer than seven alcoholic beverages per week), and current heavy drinker (more than seven alcoholic beverages per week).^38^ Physical exercise was measured based on a question asking how often the respondent participated in “sports or activities that are moderately energetic such as gardening, cleaning the car, walking at a moderate pace, dancing, and floor or stretching exercises.” Response categories included hardly ever/never (reference), one to three times a month, once a week, more than once a week, and every day.

#### Covariates

We controlled for basic sociodemographic covariates, including age (in years), gender (0=men, 1=women), and race/ethnicity (non-Hispanic white [reference], non-Hispanic black, Hispanic, and other).

### Statistical analyses

We estimated a series of ordinal logistic regression models to examine the relationship between sexual identity and self-rated health using the progressive adjustment method.^5,39,40^ Model 1 only included sexual identity without controlling for any covariates. Model 2 controlled for basic sociodemographic covariates, including age, gender, and race/ethnicity. Model 3–5 added marital status, SES, and health behaviors separately. Model 6 included all covariates.

As our analytic sample included only those who answered the question on sexual identity, we applied an approach developed by Heckman to adjust for the sample selection bias in all regression models.^41^ This approach consists of modeling the probability of not answering the sexual identity question using logistic regression, conditional on a set of predictors (e.g., age, gender, race-ethnicity, marital status, education). Then, for individuals who answered the questions on sexual identity and thus included in our analytic sample, self-rated health was modeled as a function of a set of independent variables plus the predicted probability of not answering the sexual identity question. All analyses were weighted using the person-level analysis weight and further adjusted for the complex survey design of HRS using the STATA SVY function.^42^

## Results

Table 1, which shows weighted descriptive statistics for all analytic variables by sexual identity, reveals significant differences in sample characteristics across sexual identity groups. Compared to heterosexual midlife respondents, gay/lesbian midlife respondents reported higher levels of self-rated health while bisexual respondents and others reported lower levels of self-rated health. Heterosexual respondents were more likely to be married than all the other sexual identity groups. Compared to heterosexual respondents, gay/lesbian respondents had higher levels of education and more household wealth, while bisexual and other sexual minority respondents had lower education and less household income. Relative to heterosexual respondents, gay/lesbian respondents were less likely to be nondrinkers, while bisexual respondents were more likely to be current smokers, but less likely to be current heavy drinkers. Moreover, heterosexual respondents were more likely than bisexual and other respondents to engage in exercise more than once a week.

**Table 1.** Weighted Descriptive Statistics for Analytic Variables

	Total	Heterosexual	Gay/Lesbian	Bisexual	Other
	*N*=3623	*N*=3418	*N*=99	*N*=38	*N*=68
Self-rated health, mean (SD)	2.44 (1.08)	2.44 (1.07)	2.71^*^ (1.00)	1.94^**^ (1.14)	2.06^*^ (1.27)
*Mediators*					
Marital status					
Married (%) (ref.)	56.75	58.18	24.36^***^	33.43^**^	37.13^***^
Cohabitation (%)	7.03	6.33	30.79^***^	5.10	10.08
Previously married (%)	22.44	22.83	6.74^***^	27.72	22.60
Never married (%)	13.79	12.66	38.10^***^	33.74^***^	30.20^***^
Socioeconomic status					
Education					
Less than high school (%) (ref.)	13.13	13.00	3.99^**^	30.16^**^	27.85^***^
High school graduate (%)	24.68	25.06	16.13^*^	9.68^*^	24.35
Some college (%)	27.78	27.64	24.30	47.66^**^	31.40
College graduate and above (%)	34.41	34.31	55.58^***^	12.49^**^	16.40^**^
Log household income	10.24 (2.19)	10.26 (2.17)	10.35 (2.66)	9.46^*^ (3.22)	9.72^*^ (1.98)
Log household wealth	13.70 (0.43)	13.70 (0.42)	13.86^*^ (0.32)	13.79 (0.26)	13.64 (0.50)
Health behaviors					
Current smoker					
No (%) (ref.)	77.90	78.07	73.56	55.67^**^	86.33
Yes (%)	22.10	21.92	26.44	44.33^**^	13.67
Current drinker					
No (%) (ref.)	47.41	47.35	37.20^*^	52.36	66.21^**^
Current light drinker (%)	34.27	34.10	43.05	41.75	25.58
Current heavy drinker (%)	18.32	18.55	19.75	5.89^*^	8.30^*^
Frequency of moderate exercise					
Hardly ever or never (%) (ref.)	12.69	12.19	7.72	41.17^***^	36.08^***^
One to three times a month (%)	11.83	11.88	14.22	6.20	7.99
Once a week (%)	17.13	17.28	14.01	23.82	9.74
More than once a week (%)	49.80	50.15	56.24	24.78^**^	30.74^**^
Everyday (%)	8.55	8.50	7.82	4.04	15.45^+^
*Demographic covariates*					
Female (%)	45.15	45.15	46.25	54.95	38.19
Race-ethnicity					
Non-Hispanic white (%) (ref.)	60.08	60.66	62.21	67.81	17.68 ^***^
Non-Hispanic black (%)	13.45	13.21	13.72	18.01	25.50^**^
Hispanic (%)	6.63	6.65	6.83	5.82	16.16^**^
Other races (%)	19.82	19.65	17.24	8.36^+^	40.66^***^
Age, mean (SD)	53.70^***^ (2.03)	53.72 (2.01)	53.38 (2.42)	54.19 (2.28)	52.86^***^ (1.84)

^***^ *p*<0.001, ^**^p<0.01, ^*^ *p*<0.05, ^+^*p*<0.10: Two-tailed *t*-tests for continuous variables and tests of proportions for categorical variables comparing gay/lesbian, bisexual, or other versus heterosexual groups.

Table 2 shows the estimated odds ratios for self-rated health from the ordinal logistic regression models. The results of Model 1 suggest that without controlling for any covariates, the odds of reporting higher categories of health (hereafter “better health”) were 56% (OR=0.44, 95% CI=0.25–0.79) lower for bisexual midlifers and 50% (OR=0.50, 95% CI=0.25–1.00) lower for other nonheterosexual identified midlifers than heterosexual midlifers; gay and lesbian respondents had higher odds of reporting better health than their heterosexual counterparts although the result was not statistically significant (OR=1.54, 95% CI=0.80–2.94).

**Table 2.** Sexual Identity Differences in Self-Rated Health from Ordinal Logit Regression Models, Health and Retirement Study 2016 (*N*=3623)

	Model 1		Model 2		Model 3		Model 4		Model 5		Model 6	
	OR	95% CI	OR	95% CI	OR	95% CI	OR	95% CI	OR	95% CI	OR	95% CI
Sexual identity (ref: heterosexual)												
Gay/lesbian	1.54	0.80–2.94	1.78^+^	0.99–3.19	1.83^*^	1.05–3.18	1.62^+^	0.93–2.80	1.73	0.89–3.35	1.56	0.84–2.89
Bisexual	0.44^**^	0.25–0.79	0.43^**^	0.25–0.76	0.61	0.33–1.12	0.61	0.33–1.11	0.63	0.31–1.27	0.76	0.35–1.64
Other	0.50^*^	0.25–1.00	0.61	0.31–1.20	0.92	0.42–2.01	0.86	0.46–1.59	0.82	0.38–1.79	1.09	0.51–2.34
Marital status (ref: married)												
Cohabiting					0.59^*^	0.37–0.94					0.96	0.55–1.71
Previously married					0.11^***^	0.07–0.18					0.34^**^	0.16–0.74
Never married					0.07^***^	0.04–0.12					0.27^*^	0.10–0.72
Socioeconomic status												
Education (ref. less than high school)												
High school graduate/GED							2.16^***^	1.67–2.80			1.35	0.92–1.99
Some college							3.49^***^	2.61–4.67			1.82^*^	1.07–3.10
College and above							7.00^***^	4.94–9.91			2.65^**^	1.36–5.16
Log of household income							1.22^***^	1.15–1.30			1.13^**^	1.05–1.21
Log of household wealth							0.86	0.59–1.26			1.22	0.80–1.86
Health behaviors												
Smoking (ref. current non-smoker)												
Current smoker									0.49^***^	0.39–0.61	0.64^***^	0.51–0.82
Drinking (ref: current non-drinker)												
Current light drinker									1.85^***^	1.38–2.47	1.54^***^	1.19–1.99
Current heavy drinker									1.51^*^	1.02–2.25	1.29	0.90–1.87
Exercise (ref: hardly ever or never)												
One to three times a month									1.64^**^	1.17–2.28	1.44^*^	1.01–2.07
Once a week									2.62^***^	1.95–3.51	2.31^***^	1.74–3.08
More than once a week									4.21^***^	3.27–5.42	3.19^***^	2.47–4.12
Everyday									4.62^***^	2.78–7.67	3.92^***^	2.42–6.37

^***^ *p*<0.001, ^**^p<0.01, ^*^p<0.05, ^+^*p*<0.10. Model 1 controlled for no other covariates. Models 2–6 controlled for age, gender, race-ethnicity, and predicted probability of not answering the sexual identity question.

GED, General Education Development.

After age, gender, and race-ethnicity were controlled in Model 2, the worse self-rated health of bisexual relative to heterosexual midlifers remained significant (OR=0.43, 95% CI=0.25–0.76), and the better self-rated health of gay and lesbian relative to heterosexual midlifers became marginally significant (OR=1.78, 95% CI=0.99–3.19). When marital status was added to Model 3, the better self-rated health of gay and lesbian relative to heterosexual midlifers became significant (OR=1.83, 95% CI=1.05–3.18), suggesting that marital status was a suppressor for self-rated health difference between gay/lesbian and heterosexual respondents. That is, if gay and lesbian midlifers had the same distribution of marital status as heterosexual midlifers, they would have even better health. However, the better self-rated health of gay and lesbian respondents relative to their heterosexual counterparts became nonsignificant after SES and health behaviors were added (Models 4–6).

For bisexual midlifers, their relative disadvantage in self-rated health was reduced to be insignificant when marital status, SES, and health behavior variables were, respectively and collectively, added to the regression (Models 3–6). Additional analysis (results not shown but available upon request) using *t*-tests suggested that changes in the coefficient for bisexual identity were statistically significant comparing Models 3–6 versus Model 2.

The results of Models 3–6 also suggest that being unmarried (cohabiting, previously married, or never married) and being a current smoker were both related to poorer self-rated health, while higher education, higher income, drinking, and more frequent exercise were all related to better self-rated health. Our further investigation of sexual identity differences in marital status, SES, and health behaviors (Table 3) suggested that bisexual midlifers were more likely to be previously or never married, had lower education and income, were more likely to smoke, and exercise less often than heterosexual midlifers—all of which are significant predictors for self-rated health (as suggested in Models 3–6 in Table 2).

**Table 3.** Sexual Identity Differences in Marital Status, Socioeconomic Status, and Health Behaviors, Health and Retirement Study 2016 (*N*=3623)

	Marital status (multinomial regression: married as the baseline category)					
	Cohabitation		Previously married		Never married	
	RRR	95% CI	RRR	95% CI	RRR	95% CI
Lesbians/gays	8.04^***^	4.13 to 15.66	0.55	0.13 to 2.22	5.59^*^	1.35 to 23.18
Bisexuals	1.91	0.72 to 5.05	4.38^+^	0.86 to 22.20	18.59^**^	3.07 to 112.46
Others	3.68^+^	0.90 to 15.06	2.55	0.47 to 13.83	6.52^*^	1.34 to 31.66

	Socioeconomic status					
	Education (ordinal logit regression)		Income (OLS regression)		Wealth (OLS regression)	
	OR	95% CI	Coef.	95% CI	Coef.	95% CI
Lesbians/gays	2.12^*^	1.16 to 3.89	0.12	−0.93 to 1.17	0.16^**^	0.05 to 0.27
Bisexuals	0.43^+^	0.16 to 1.17	−0.79^+^	−1.72 to 0.15	0.08	−0.04 to 0.21
Others	0.51^*^	0.27 to 0.98	−0.19	−0.72 to 1.49	0.01	−0.14 to 0.17

	Health behaviors					
	Current smoker (binary logit regression)		Current drinker (ordinal logit regression)		Exercise (ordinal logit regression)	
	OR	95% CI	OR	95% CI	OR	95% CI
Lesbians/gays	1.04	0.53 to 2.07	1.25	0.72 to 2.19	1.13	0.71 to 1.80
Bisexuals	2.66^+^	0.93 to 7.64	0.65	0.26 to 1.66	0.27^***^	0.11 to 0.65
Others	0.50^+^	0.23 to 1.09	0.50^+^	0.24 to 1.08	0.53	0.13 to 2.21

^***^ *p*<0.001, ^**^p<0.01, ^*^p<0.05, ^+^*p*<0.10. Model 1 controlled for no other covariates. Models 2–6 controlled for age, gender, race-ethnicity, and predicted probability of not answering the sexual identity question.

OLS, ordinary least squares; RRR, relative risk ratios.

## Discussion

This study is one of the first to use nationally representative data to examine health disparities among U.S. sexual minority midlifers. We found that bisexual midlifers reported significantly worse health than heterosexual midlifers. This result is partially consistent with Hypothesis 1 as well as an emerging literature suggesting that bisexual people experience the most serious health disadvantages among the LGB population.^29–31^

The current study extends the evidence for bisexual health disadvantages to the midlife stage and supports previous findings showing that bisexual people report the highest level of mental distress compared to other major sexual identity groups in middle adulthood.^8^ Relative to gay/lesbian and heterosexual people, bisexual people may experience unique negative stereotypes, for example, the perception that they are confused or indecisive about their sexual identity, are less able to commit to the values and norms of either the heterosexual or gay/lesbian community, and are less trustworthy in romantic relationships.^30,43–46^ Because prejudice against bisexuality can come from both the heterosexual and gay/lesbian communities, bisexual individuals experience a “double stigma” that may have more detrimental effects on health.^47^

Moreover, we found that marital status, SES, and health behaviors (in particular smoking and exercising) explained most of self-rated health disadvantages among bisexual midlifers relative to their heterosexual counterparts; this finding supports Hypothesis 2. The current analysis confirmed the long-standing finding that married people report better health than unmarried people.^15^ The lower likelihood of being married and lower SES among bisexual midlifers (relative to their heterosexual counterparts) contribute to their poorer health. As prior studies have suggested, due to the bisexual stigma that lowers partnership rates and selectivity, bisexual people may face more difficulties finding partners, particularly those with more favorable SES.^30,43^ Our analysis also confirmed the health effects of smoking and exercising, and showed that differences in smoking and exercising accounted for the self-rated health disparity between bisexual and heterosexual midlifers.

We did not find evidence of a self-rated health disadvantage for gay and lesbian midlifers relative to their heterosexual counterparts. Indeed, with age, gender, race/ethnicity, and marital status held constant, gay and lesbian midlifers reported better health than heterosexual midlifers. This result was partially inconsistent with Hypothesis 1, but it aligns with a small number of recent studies showing no health disadvantage for gays/lesbians relative to their heterosexual counterparts.^8,29,30^ Some scholars have argued that the societal climate has steadily become more accepting of sexual minority people, in particular gay and lesbian people^48–51^; this shift may have engendered improvements in social support and health among gay and lesbian people.

This study has several limitations. First, sexual orientation encompasses multiple dimensions, including identity, attraction, and behavior. Currently, HRS includes only one measure of sexual identity, and thus may undercount sexual minority individuals, especially those in midlife and older age who do not openly identify as gay, lesbian, or bisexual. In addition, those who experience more sexuality-based discrimination may be less likely to disclose their sexual identities in the survey context. This could bias our results by underestimating the health disparity between sexual minority and heterosexual people.

Second, the HRS sampling is not designed to oversample or represent sexual minorities, and sample sizes for LGB groups are small in our data. This may lead to low statistical power and increase the risk of type II error. The low statistical power related to small sample size may explain some of the nonsignificant findings in our study. Moreover, the small sample size also limits our ability to further examine differential experiences among sexual minorities (e.g., gay men vs. lesbian women, bisexual men vs. bisexual women). Future research should explore heterogeneous experiences among sexual minority groups if sample sizes allow.

Third, although we ideally would have addressed our research question using causal mediation methods, our analysis is based on cross-sectional data that limit our ability to fully determine causality.

Fourth, although we applied the Heckman correction to adjust for the sample selection bias due to skipping the sexual identity question, selection bias stemming from other sources (e.g., loss to follow-up over time in the cohort) may still be present.

Finally, our measurements are limited. Despite its strong predicting power for mortality,^34,35^ self-rated health is a subjective measure and may not fully reveal health disparities faced by sexual minorities. Because the HRS only asked about sexual identity to a new refreshment sample of adults in 2016, we are unable to consider the history of health and health behaviors into our analysis.^35^ Also, the challenges and minority stressors that sexual minority individuals face in midlife are more complex than the measures used in the current analysis. Future research should consider examining how other minority stressors (e.g., discriminative laws and policies) shape the health trajectories of sexual minority individuals over the life course.

## Conclusion

Midlife comprises a critical life course stage—many middle-aged adults experience major life stressors that can be detrimental to health.^2,4^ A minority stress perspective suggests that sexual minority midlifers likely face additional barriers and challenges that may hurt their health. Yet, few previous studies have focused on the health of sexual minority individuals in midlife. We documented a significant health disadvantage among bisexual midlifers but not gay/lesbian midlifers relative to their heterosexual counterparts; this pattern highlights the diversity of the sexual minority population in midlife. Future studies should further explore the specific pathways that lead to such health disparities, with attention to the roles of relationships, SES, and health behaviors in sexual minority communities and health.

## Abbreviations Used

HRS: Health and Retirement Study
LGB: lesbian, gay, and bisexual
OLS: ordinary least squares
RRR: relative risk ratios
SES: socioeconomic status
